# Time Perception and Dynamics of Facial Expressions of Emotions

**DOI:** 10.1371/journal.pone.0097944

**Published:** 2014-05-16

**Authors:** Sophie L. Fayolle, Sylvie Droit-Volet

**Affiliations:** Blaise Pascal University, CNRS, UMR 6024, Clermont-Ferrand, France; National University of Singapore, Singapore

## Abstract

Two experiments were run to examine the effects of dynamic displays of facial expressions of emotions on time judgments. The participants were given a temporal bisection task with emotional facial expressions presented in a dynamic or a static display. Two emotional facial expressions and a neutral expression were tested and compared. Each of the emotional expressions had the same affective valence (unpleasant), but one was high-arousing (expressing anger) and the other low-arousing (expressing sadness). Our results showed that time judgments are highly sensitive to movements in facial expressions and the emotions expressed. Indeed, longer perceived durations were found in response to the dynamic faces and the high-arousing emotional expressions compared to the static faces and low-arousing expressions. In addition, the facial movements amplified the effect of emotions on time perception. Dynamic facial expressions are thus interesting tools for examining variations in temporal judgments in different social contexts.

## Introduction

Researchers are becoming increasingly aware of the importance for humans of time and its processing in different contexts and especially in the context of social interaction. Indeed, the flexibility of temporal behaviors in social interaction is an indicator of the efficiency of social adaptation, [1], [2], [3]. An individual who is unable to anticipate the actions of others, who always responds too late or too early, is socially inept. According to studies of facial expressions, emotions perceived in others play a critical role in making it possible to understand their behavioral intentions. One important component of the perception of facial expressions is indeed the readiness to act in order to enter into a relationship with others, [4], [5], [6]. Action readiness is defined as the predisposition of individuals to engage in a social relationship with others by either approaching or avoiding them, [7]. When somebody expresses anger, and might therefore become aggressive, there is an automatic readiness to act defensively in order to ensure survival, [8]. The arousal level increases, the pupils dilate, the heart accelerates, and the muscles contract. The whole body is mobilized to be ready to act as quickly as possible, i.e., to escape or to attack. In contrast, there is less urgency for action when confronted with a sad person, although it is also important to act in order to comfort or help this person, [9].

A number of recent studies in psychology have used a temporal bisection task to investigate the effect of facial expressions on time judgments, [10], [11], [12], [13], [14], [15], [16]. In this task, participants are presented with a short (*S*) and a long (*L*) standard duration in the form of a simple oval. In a testing phase, they are then presented with comparison durations of similar or intermediate lengths. Their task is to judge whether the comparison durations are more similar to *S* or *L*. However, in the testing phase, the comparison durations are presented in the form of emotional facial expressions. The results have shown distortions in temporal judgments of emotional expressions compared to neutral expressions, [17], [18]. Nevertheless, the type of temporal distortion depends on the emotion perceived. When the faces express a secondary emotion of shame, a shortening effect occurs, with the presentation duration of ashamed faces being judged shorter than that of neutral expressions, [19]. Ogden [20] also showed that the perception of attractive faces shortens subjective time. This shortening effect is explained by the fact that these facial expressions divert attention away from the processing of time. According to the internal clock models, [21], [22], [23], the representation of time is determined by the number of temporal units (pulses) emitted by a pacemaker-like system, and accumulated during the presentation of a stimulus. Temporal units are therefore lost when attention is distracted away from the processing of time. In contrast, a lengthening effect occurs in response to the expressions of anger or fear, [10], [12], [14], [15]. Within the framework of the internal clock models, this lengthening effect has been explained in terms of the increase in the arousal level induced by the perception of the emotions of anger and fear which speed up the internal clock mechanism underlying the representation of time: The greater the number of temporal units, the longer the stimulus duration is judged to be. As Droit-Volet and Meck [13] have argued, this time dilatation results from the speeding up of the internal clock mechanism, one of the main functions of which is to allow subjects to prepare for action quickly. This effect has been observed in children as young as 3 years of age, thus suggesting that this is an automatic process, [24]. The extent of this subjective lengthening of time in response to the perception of facial expressions has nevertheless been found to be smaller for the emotions of happiness and sadness (and even absent in the case of sadness) than for those of anger and fear, [12], [13], [14]. To explain these results, the authors suggested that happy and sad faces are less arousing and that the rate of acceleration of the internal clock system is therefore less rapid.

However, even though studies of time perception in response to facial expressions have revealed significant effects on time judgments, these are still relatively weak, even in the case of high-arousing facial expressions such as the expression of anger. Like most studies of facial expressions, these studies have used photographs of static faces expressing intense emotion. However, if the understanding of the behavioral intentions of others is important for action readiness and its effect on the internal clock mechanisms, we can suppose that the perception of moving faces will increase emotional effects on time judgments. Indeed, individuals habitually make use of their perception of other people's movements to predict their behavior, [25]. As we discuss later, several studies have shown that the emotions of moving faces are judged to be more intense and realistic than those of static faces, and that the dynamic display of facial expressions enhances the accuracy of emotion recognition, [26], [27], [28], [29]. The dynamic aspects of facial behaviors have been neglected in studies of facial expressions [30] and have never been investigated in the time perception field. Our study is therefore original in attempting to examine the influence of dynamic aspects of emotional facial expressions on the perception of time. Our question is: are the distortions in judgments of time in response to emotional expressions greater when facial expressions are presented dynamically (morph movie) than when they are presented statically? Two experiments were run in our study. The first experiment tested the effect on time perception of movements in facial expression and the emotions expressed. In a temporal bisection task, the participants had thus to judge the presentation duration of emotional facial expressions presented either in a dynamic or a static display in comparison to a neutral facial expression, i.e., with no facial movement. Two emotional facial expressions were tested (anger and sadness) because they had the same affective valence (unpleasant), but one was considered as high-arousing (expressing anger) and the other as low-arousing (expressing sadness). The affective dimension and the arousal level induced by the perception of dynamic and static facial expressions were also assessed using the Self-Assessment Manikin scale (SAM), [31]. The second experiment compared these two emotional facial expressions (anger *vs.* sadness) when they were presented dynamically in the same bisection task or statically.

## Experiment 1

### Method

#### Participants

The participants consisted of 104 undergraduate psychology students at Blaise Pascal University (Clermont-Ferrand, France). They received a course credit and signed a formal agreement to participate in this experiment which was approved by the Clermont-Ferrand Sud-Est VI Statutory Ethics Committee (Comité de protection des Personnes (CPP) Sud-Est 6, France) according to the articles of law L. 1121-1-2 and R 1121-3.

#### Materials

The participants were tested individually in a quiet room and were seated 50 cm from a PC screen. An E-prime program (1.2. psychology Software Tools, Pittsburgh, PA) controlled the experiment and recorded the data. Participants gave their responses by pressing two keys on the computer keyboard, “d” (response “Short”) and “k” (response “Long”), with the button-press assignment being counterbalanced across subjects. During the training phase, the stimulus to be timed was an oval with a mottled texture (white, gray, black) presented on a black background in the center of the computer screen. During the testing phase, the temporal stimuli consisted of faces presented on the same black background. These were taken from a validated set of images ranging from a neutral to an intense emotional expression for different facial identities, [32]. The faces of three different women expressing the emotions of anger and sadness as well as a neutral expression were chosen for our experiment. The image of the most intense facial expression (150% intensity) was presented during the tested duration stimulus in the static condition. In the dynamic condition, in order to generate a dynamic emotional expression from a set of pictures morphed from the neutral to the most intense emotional expression (150 % intensity), a sequence of 17 different images of 12 ms each was used. These images increased in emotional intensity (0%, 5%, 10%, 20%, 30%, 40%, 50%, 60%, 70%, 80%, 90%, 100%, 110%, 120%, 130%, 140%, 150%) in accordance with Benson and Perrett's morphing technique, [33]. The facial movement took 204 ms in each dynamic presentation and was followed by the continued display of the image corresponding to the most intense facial expression used in the static condition. The presentation duration of this last image depended on the tested duration. For the neutral expressions, which by definition did not involve a facial movement (no-facial expression), we decided to use the same images of neutral faces in both the dynamic and the static condition.

#### Procedure

The participants were assigned to one of the four experimental groups: (1) dynamic-anger, (2) dynamic-sadness, (3) static-anger and (4) static-sadness. In each group, the participants were presented with a neutral facial expression and an emotional facial expression (anger or sadness), with the presented emotional expressions being either dynamic or static depending on the experimental group. The procedure was similar in the 4 experimental groups. The participants were initially trained to respond “short” or “long” on 10 trials after experiencing the short (0.4 s) and the long (1.6 s) standard duration (5 trials per duration) presented in the form of an oval. They were then presented with 7 different comparison durations (0.4, 0.6, 0.8, 1.0, 1.2, 1.4 and 1.6 s) presented in the form of neutral or emotional facial expressions. The participants' task was to judge whether the comparison duration was more similar to the “short” or the “long” standard duration. Each participant completed 9 trials for each comparison duration presented with the neutral and the emotional facial expression (7×2). The 9 trials consisted of 3 trials for each of the 3 female faces. The total number of trials was thus 126 trials presented in a random order in each block of 42 trials (3 females faces ×7 comparison durations ×2 facial expressions). Each trial started when the participant pressed the space bar after seeing the word “ready!” displayed on the screen after an inter-trial interval which was randomly chosen between 0.5 s and 1.0 s.

At the end of the experimental session, the participants used the 9-point scale of the Self-Assessment Manikin (SAM) [31] to rate the neutral and emotional expression of the three faces they had seen in the bisection task: (1) for their affective valence - from unpleasant to pleasant, and (2) for their arousal level - from low-arousal to high-arousal. The presentation duration of the facial expressions was at the mid-point between the short and the long standard durations, i.e., 1.0 s. In each group, the participants therefore rated 6 different emotional stimuli presented in a random order (3 faces ×2 emotions).

### Results

#### Emotional assessment of facial expressions

**Figure 1:**
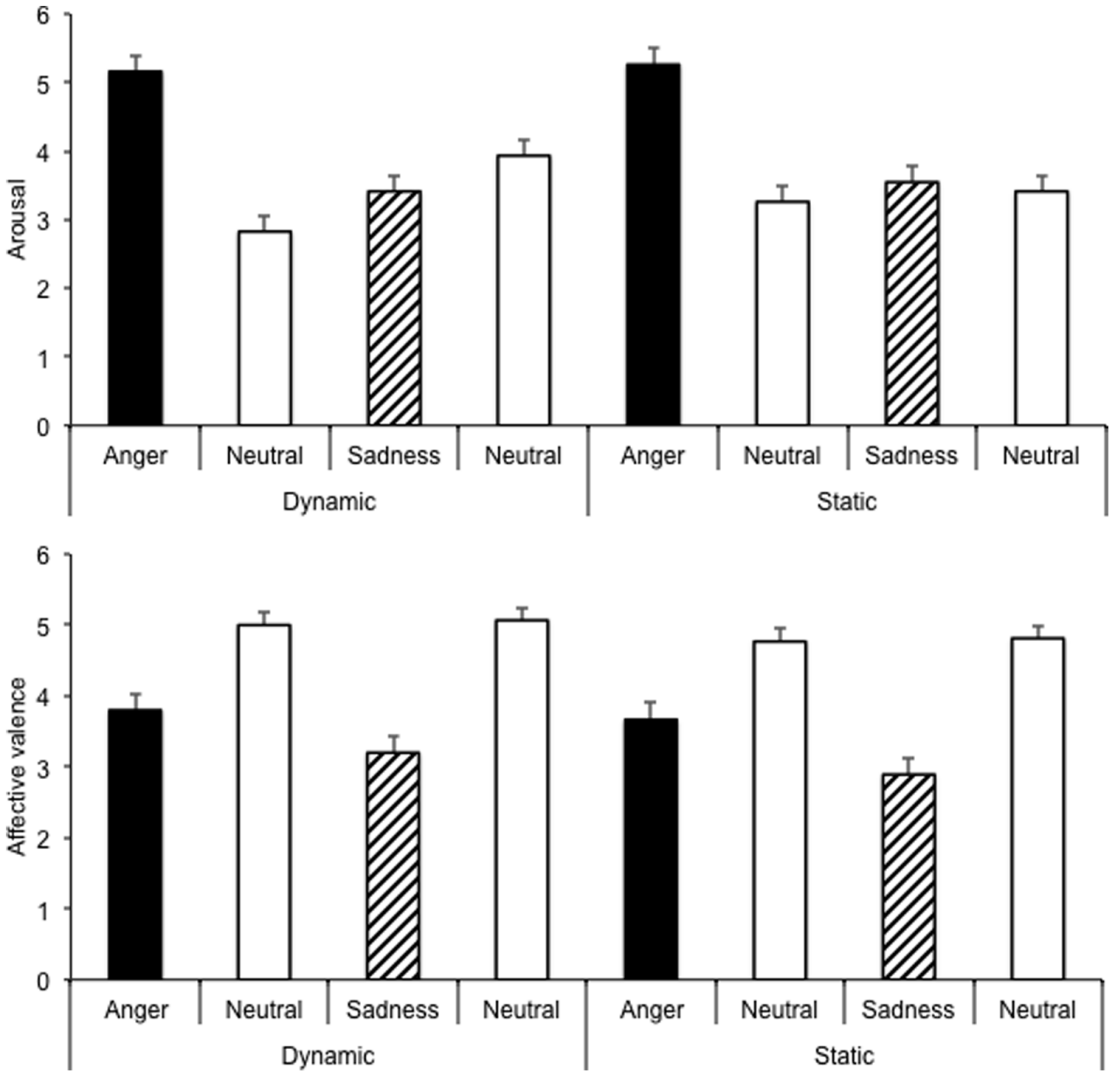
Arousal and affective valence ratings of neutral and emotional facial expressions presented in a dynamic and a static display.

Figure 1 shows the emotional ratings for the different emotional and neutral facial expressions used in the four experimental groups when the emotional facial expressions (results averaged over the three female faces) were presented in a dynamic and a static display. An ANOVA was run on arousal and affective valence ratings with the emotion (neutral *vs.* emotional expression) as a within-subjects factor and the experimental group as a between-subjects factor. The results of 6 out of the 104 participants were not included because they did not respond to these scales. The ANOVA on arousal showed a significant main effect of emotion, *F* (1, 94)  = 65.85, *p* = .0001, and group, *F* (3, 94)  = 3.08, *p* = .03, as well as a significant emotion x group interaction, *F* (3, 94)  = 33.14, *p* = 0001. The analyses for each experimental group taken separately revealed that the facial expressions of anger were systematically judged more arousing than the neutral expression both in the dynamic and the static condition (Bonferroni tests, both *p*<.05). For the expressions of anger, no significant difference in arousal was observed between the dynamic and the static presentation (Bonferroni test, *p*>.05). In contrast, the facial expressions of sadness tended to be judged as low-arousing when presented dynamically but not statically, with the sad expressions being rated as less arousing than the neutral expressions in the dynamic condition (*p*<.05), and similarly low-arousing in the static condition (*p*>.05). As would be expected because the neutral stimuli were identical in the two conditions, no difference was observed in the arousal judgments for the neutral expressions presented in the static and the dynamic condition.

The ANOVA on the affective valence ratings also showed a significant main effect of emotion, *F* (1, 94)  = 144.72, *p* = .0001, and a significant emotion x group interaction, *F* (3, 94)  = 2.89, *p* = .04, while the main effect of group did not reach significance, *F* (3, 94)  = 1.95, *p* = .13. The significant interaction between emotion and group indicated that the participants systematically judged the angry and sad expressions to be less pleasant than the neutral expressions (Bonferroni tests, both *p*<.05). However, no difference between groups (dynamic *vs.* static) was found for the neutral expressions or for the sad or the angry expressions (Bonferroni, all *p*>.05).

In sum, as the self-assessment of emotions induced by the perception of facial expressions suggests, the angry expressions were judged to be more arousing than the sad expressions, whereas their affective valence was judged similar. The static-dynamic mode of presentation of the images did not change the arousal ratings, except in the case of the sad expressions, which were judged to be less arousing than the neutral expressions in the dynamic condition.

#### Temporal performance

**Figure 2:**
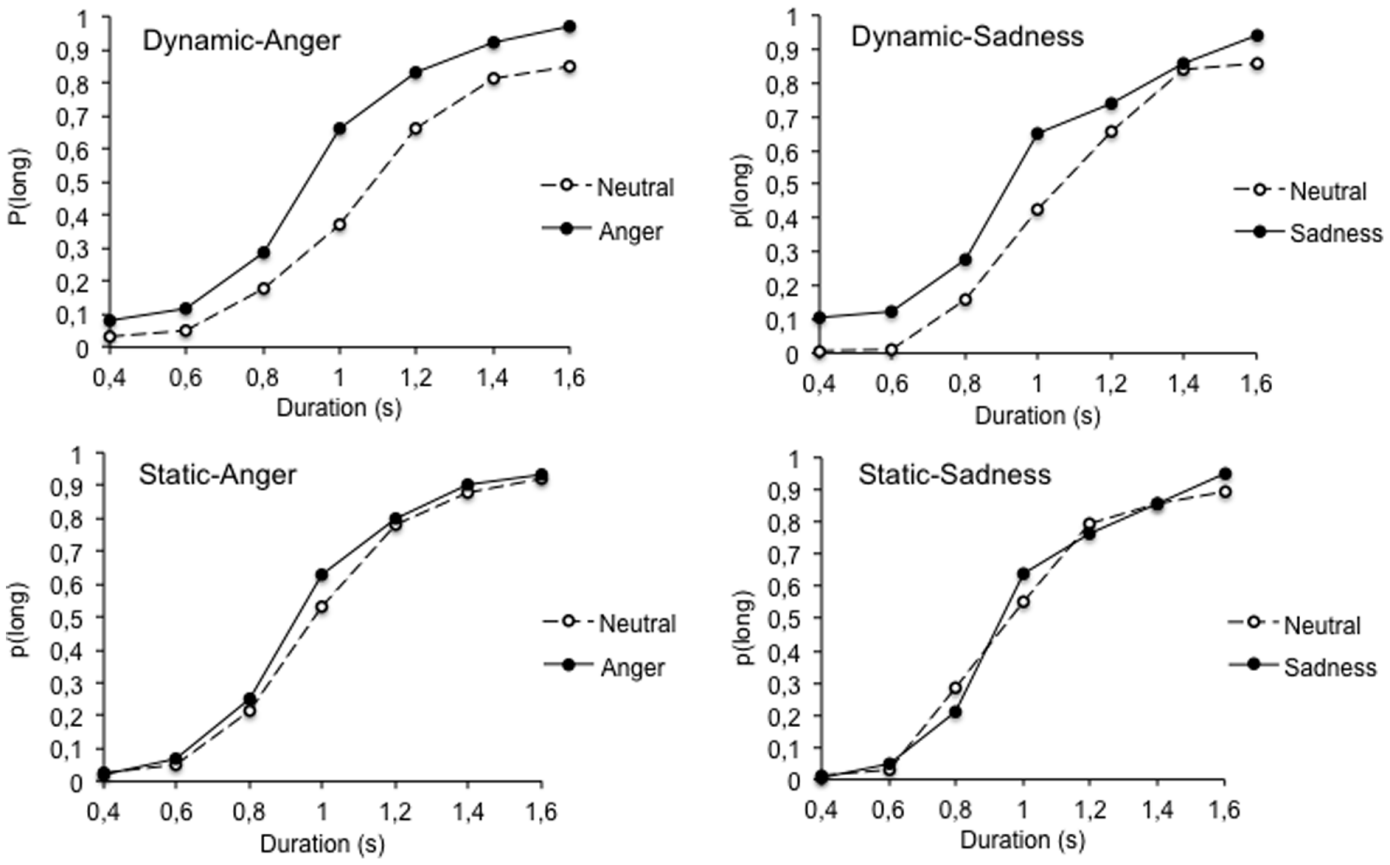
Proportion of long responses plotted against the comparison durations (s) for the neutral facial expression and the emotional (anger or sadness) facial expressions presented in a dynamic and a static display.

Figure 2 presents the psychophysical functions with the proportion of long responses - *p*(long) - plotted against the comparison durations in the 4 experimental groups. This figure reveals an important effect of the dynamic features of facial expressions on time judgments. Indeed, irrespective of the emotion in question, the psychophysical functions shifted toward the left for the emotional expressions presented dynamically compared to the neutral expressions (i.e., with no facial expression). In addition, the magnitude of this leftward shift seems to have been larger for the angry than for the sad expressions. An initial ANOVA run on *p*(long) revealed a significant interaction between emotion and experimental group, *F*(3, 100)  = 11.79, *p* = .0001. Therefore, in order to account better for variations in the shape of the psychophysical functions, we calculated two temporal parameters: the Bisection Point (BP) and the Weber Ratio (WR) (Table 1). The BP is the point of subjective equality, that is the stimulus duration for which subjects respond long as often as short, *p*(long) = .50. The WR is the difference limen - (D(*p*(long)  = .75) - D(*p*(long)  = .25)) /2 - divided by the BP. This is an index of time sensitivity: The lower the WR, the steeper the psychophysical function and the higher the temporal sensitivity. These two parameters were derived from the significant fit of the individual data with the pseudo-logistic function (mean *R* ^2^  = .95, *ES* = .005, *p*<.05), which provided good fits for the bisection data in different conditions, [34], [35]. For 4 out of 104 participants, this pseudo-logistic fit was not significant because their bisection curves were too flat or not orderly. The results for these 4 participants were thus excluded from the subsequent statistical analyses. The ANOVA on the WR did not show any significant results (all *p*>.05), thus suggesting that time sensitivity did not change with the type of emotional expression and associated presentation modality (static *vs.* dynamic). In contrast, the ANOVA on the BP revealed a significant main effect of emotion, *F*(1, 96)  = 40.72, *p* = .0001, and a significant interaction between emotion and group, *F*(3, 96)  = 6.34, *p* = .001, while the effect of group did not reach significance, *F*(3, 96)  = .65, *p* = .58. As Table 1 indicates, when the presentation of facial expressions was static, the BP was lower for the angry than for the neutral expressions (Bonferroni, *p*<.05), whereas it was similar for the sad and the neutral expressions (*p*>.05). In line with the results of previous studies, this confirms that a lengthening effect occurred when the participants were presented with high-arousing facial expressions (anger). When the facial emotional expressions were presented dynamically, a temporal lengthening effect was observed for all emotional expressions (high- or low-arousing) compared to the neutral expression with no facial movement. Indeed, the BP was lower for the emotional expressions (anger or sadness) than for the neutral expressions (Bonferroni, both *p*<.05). However, contrary to what is suggested in Figure 2, the difference in the contrast between the BP values for the neutral and angry expressions, on the one hand, and the neutral and sad expressions, on the other, did not reach significance (*p*>.05). This indicates that facial movements have a major influence on time judgment, independently of the nature of the emotions perceived. This important effect of facial movements on time perception (with the dynamic faces associated with the emotional expressions and the no-dynamic faces associated with the neutral expression) have probably masked the effects of emotions per se on time perception. Therefore, a second experiment was carried out using 2 emotional expressions (anger and sadness) presented dynamically in the same bisection task (group 1). The same emotional faces were also presented in the static condition for comparison (group 2).

**Table 1:** Mean (Error Standard) Bisection Point and Weber Ratio for the neutral facial expression and the emotional facial expressions presented in a dynamic and a static display.

	Bisection Point				Weber Ratio			
	Dynamic		Static		Dynamic		Static	
	M	*ES*	M	*ES*	M	*ES*	M	*ES*
Anger	907	*30*	921	*30*	.15	.*02*	.18	.*02*
Neutral	1074	*31*	978	*31*	.18	.*02*	.16	.*02*
Sadness	927	*30*	972	*31*	.21	.*02*	.19	.*02*
Neutral	1069	*31*	979	*32*	.18	.*02*	.19	.*02*

## Experiment 2

### Method

#### Participants, Material and Procedure

Eigthy-four new undergraduate students participated in this experiment under the same conditions as described above. These participants thus received a course credit and signed a formal agreement to participate in this second experiment which was also approved by the Sud-Est VI Statutory Ethics Committee (CPP), France.

The material and the procedure were also similar to those used in Experiment 1, except that the participants were assigned to one of two groups (dynamic *vs.* static display), with the angry and the sad expressions being presented in the same bisection task. The sad and angry expressions were thus displayed dynamically to the dynamic group, and statically to the static group.

### Results

#### Emotional assessment of facial expressions

Table 2 shows the participants' ratings of facial expressions in terms of arousal and affective valence. In line with the results of Experiment 1, the ANOVA performed on arousal with emotion and group as factors revealed a main effect of emotion, *F*(1, 82)  = 96.47, *p* = .0001, indicating that the angry faces (*M* = 4.95, *ES* = .14) were judged to be more arousing that the sad faces (*M* = 3.43, *ES* = .12). The main effect of group and the emotion x group interaction were not significant (*F*(1, 82)  = 2.39 and 0.01, respectively, *p*>.05), indicating that the movement did not change the assessment of the arousal level of emotional facial expressions. There was also a main effect of emotion on affective valence, *F*(1, 82)  = 12.65, *p* = .0001, with no other significant effect being observed, thus demonstrating that the sad faces were judged less pleasant than the angry faces.

**Table 2:** Mean (Error standard) arousal and affective valence for facial expressions of sadness and anger presented in a dynamic and a static display.

	Arousal		Affective Valence	
	M	*ES*	M	*ES*
Dynamic				
Sadness	3.26	.*17*	3.42	.*17*
Anger	4.79	.*20*	3.78	.*20*
Static				
Sadness	3.60	.*17*	3.07	.*17*
Anger	5.10	.*20*	3.66	.*20*

#### Temporal performance

**Figure 3:**
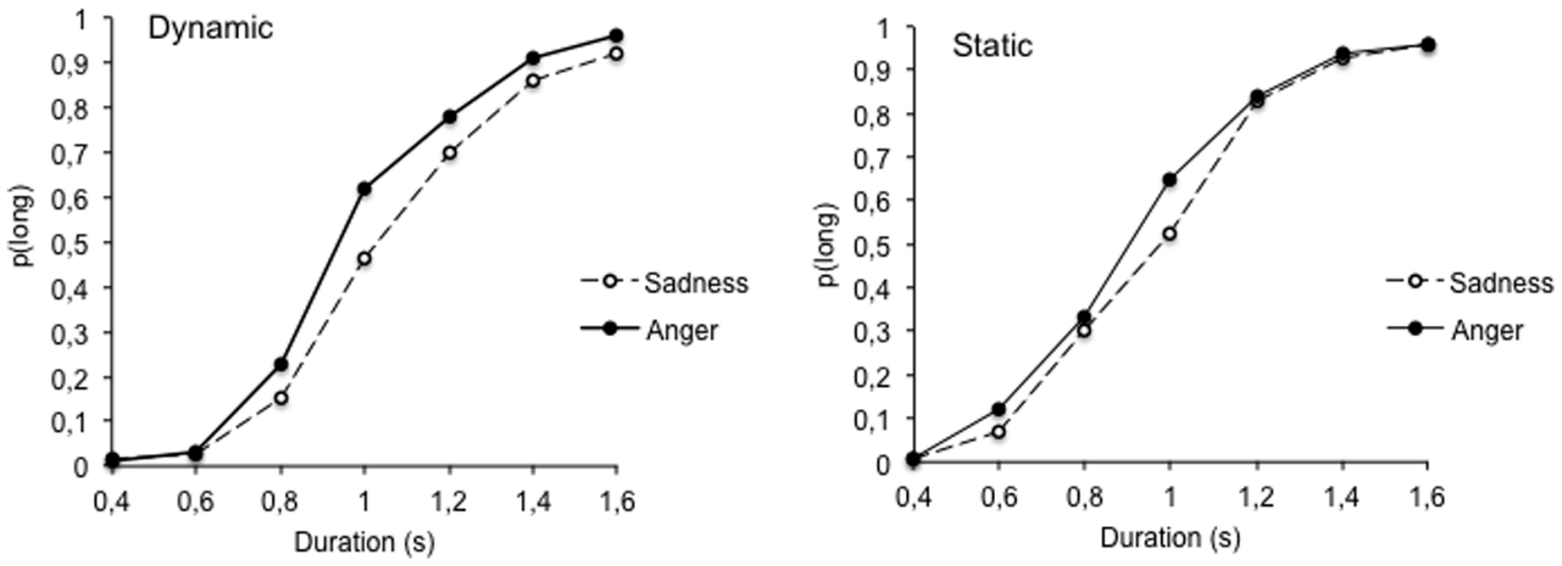
Proportion of long responses plotted against the comparison durations (s) for the facial expressions of anger and sadness presented in a dynamic and a static display.

An examination of the psychophysical functions in Figure 3 suggests that the effect of emotional facial expressions on time judgments did not disappear when the two types of emotional expressions (low- and high-arousal) were displayed dynamically in the same bisection task. On the contrary, the dynamic display of facial expressions seems to amplify the differences between the angry and the sad expressions relative to the static display of the same expressions. As in the case of Experiment 1, an ANOVA was conducted on the BP and the WR derived from the fit of the individual functions with the pseudo-logistic function that provided a good fit with our data (mean *R* ^2^ = .95, *ES* = .007, *p*<.05) (see Table 3). The ANOVA on the WR showed neither a main effect of emotion, *F*(1, 82)  = 0.76, *p* = .39, and group, *F*(1, 82)  = 1.63, *p* = .21, nor any interaction between emotion and group, *F*(1, 82)  = 0.94, *p* = .33. The ANOVA on the BP revealed a significant main effect of emotion, *F*(1, 82)  = 42.43, *p* = .0001, which clearly demonstrates that the presentation duration of faces was judged longer for the highly arousing emotion of anger than for the less arousing emotion of sadness. In addition, both the main effect of group, *F*(1, 82)  = 5.36, *p* = .02, and the group x emotion interaction were significant, *F*(1, 82)  = 5.37, *p* = .02. There was indeed a significant effect of emotion in the dynamic, *F*(1, 41)  = 46.16, *p* = .001, as well in the static condition, *F*(1, 41)  = 7.62, *p* = .01. However, the significant interaction indicated that the magnitude of the difference in the BP between the sad and the angry faces was larger in the dynamic than in the static condition, *F*(1, 82)  = 5.39, *p* = .02. This result demonstrated that the difference between the time judgments elicited for the high-arousing and the low-arousing emotional expressions was bigger for moving faces than for static faces. In other words, the perception of movements in facial expressions increases the emotional effects on time judgments which are already observed with static images.

**Table 3:** Mean (Error Standard) Bisection Point and Weber Ratio for the perception of emotional facial expressions presented in a dynamic and a static display.

	Bisection Point				Weber Ratio			
	Dynamic		Static		Dynamic		Static	
	M	*ES*	M	*ES*	M	*ES*	M	*ES*
Anger	952	*24.52*	899	*24.52*	.16	.*01*	.15	.*01*
Sadness	1048	*25.64*	944	*25.64*	.18	.*01*	.15	.*02*

## Discussion

The results of the present study using the temporal bisection task showed that the psychophysical functions were shifted toward the left for the angry expressions compared to the neutral expressions. This shift was accompanied by a significant lowering of the bisection point which indicates that time was judged longer in response to angry faces. In contrast, no difference in time judgments was observed between the sad and the neutral expressions. The self-assessment of arousal level induced by the perception of facial expressions indicated that the angry faces were judged as more arousing than the sad and the neutral faces, with no significant difference being observed between these latter two expressions. Consequently, we can conclude that the lengthening effect obtained for angry faces compared to the sad or the neutral faces was related to the increase in subjects' arousal levels. This finding is entirely consistent with the results of previous studies using emotion facial expressions, but also with those of studies using other high-arousal emotional stimuli, such as emotional pictures from the international affective pictures system (IAPS) [36], [37], [38], [39], [40], sounds from the International Affective Digital Sounds (IADS) [41], [42], or musical pieces [43]. Recently, Gil and Droit-Volet [37] manipulated the arousal level of emotional pictures (IAPS) depicting different discrete emotions (disgust, fear) and obtained evidence in support of the fundamental role of physiological activation in the way emotions influence the perception of time by leading individuals to judge emotional stimuli to be longer than neutral stimuli. However, the mechanisms underlying this emotional lengthening effect are still a matter of debate, [18]. Most researchers have suggested that the increase of physiological activation under the effects of high-arousal emotions is associated with the speeding up of brain mechanisms (pacemaker, temporal oscillators) that underlie the representation of time, [10], [12], [42]. In other words, an increase in arousal would speed up the internal clock rate, i.e., the rate at which pulses are accumulated. However, other researchers have suggested that the increase in arousal level is associated to a focus of attention on the processing of the duration of presented stimuli [39]. Indeed, when the arousal level increases in the case of a threatening situation, individuals not only prepare to act (by attacking or fleeing), but also pay more attention to potentially dangerous incoming stimuli [44]. It is therefore difficult to dissociate these mechanisms (arousal and attention) which are closely related. Whatever the case, our results provide additional data demonstrating that the perception of faces expressing a high-arousal emotion lengthens the perceived duration.

More interestingly, the originality of our study lies in showing that the perception of facial movements increased the temporal lengthening effect obtained with high-arousal emotional facial expressions. More precisely, the results of Experiment 1, comparing static with dynamic faces revealed that, per se, the perception of facial movements affects the perception of time. The moving faces were indeed judged to have been presented for longer than the static faces, irrespective of the emotion expressed by these faces. The psychophysical functions shifted toward the left and the BP was lower for the moving faces than for the static faces. However, the results of Experiment 2 relating to the effects of facial expressions of anger and sadness in the same bisection task, and using the same dynamic display, demonstrated that the dynamic presentation of facial expressions amplifies the lengthening effect linked to emotion. As far as the effect of facial movements on time judgments is concerned, our study is the first to show major distortions in such judgments in response to facial movements. However, these results with social stimuli replicate the findings of earlier studies testing the effects of stimulus motion on perceived time with different non-social stimuli (dots, rotation of sphere), [45], [46], [47]. The influence of motion on perceived time has indeed been a familiar phenomenon ever since the works of Piaget [48] and Fraisse [49] who identified time distortions in children and adults when viewing moving stimuli. For instance, in a temporal bisection task, Beckmann and Young [45] showed that the duration of a film of a rotating sphere was perceived to be longer than that of a static sphere, and that the BP value decreased with the increase in rotation speed. The question raised here is: what produces this lengthening effect with moving stimuli? The response is far from clear. It could be suggested that the perception of dynamic stimuli increases the rate of the pacemaker mechanism described in the internal clock models [50]. However, testing a series of models, Beckmann and Young [45] observed that the pacemaker-related parameters did not sufficiently capture the effects of stimulus dynamics on perceived time. They thus assumed that motion enhances the way participants track time. More recently, Matthews, Stewart and Wearden [51] referred to this idea to explain their own results on the influence of context (i.e., the difference between stimulus and background) in which a stimulus is presented on the perception of its duration. They thus argued that the flow of pulses into the accumulator during the processing of time depends on the amount of attention paid to the stimulus, with a greater amount of attention paid to the stimuli when they are more different from the background context. The flow of pulses would thus be greater in response to moving than to static stimuli because more attention is focused on dynamic changes. Consequently, one may suppose that the tracking of time would be better for moving faces than for static faces, and that this would be true for all dynamic stimuli.

However, as reported above, our results not only showed a significant main effect of facial movements on time perception, but also a significant interaction between facial movements and expressed emotions (Experiment 2). In other words, the dynamic presentation of faces increased the differences between the perceived duration of emotions. Wang and Yi Jiang [52] observed a greater dilatation in time judgments in bisection when using a motion sequence produced by a point-light walker compared to a non-biological motion or a static picture with the same number of point lights. Their results suggest that the influence of motion on human beings' temporal judgments is greater in the case of biological motion. In our study, the type of dynamic stimuli used, which consisted of a facial movement followed by a static expression did not constitute a genuinely ecological situation. Nevertheless, and in line with Wang and Yi Jiang's [52] findings, our data on facial expressions suggest that humans' time judgments are sensitivity to movements produced by other people. Most studies of facial expressions have shown that dynamic information is beneficial for the processing of emotional expressions, [30]. Dynamic displays of facial expressions do indeed improve performance on emotion recognition tasks compared to static displays of the same facial expressions, [53], [29]. However, as Krumhuber et al. [30] have argued, the beneficial effects of dynamic information are greater when static information is inefficient or unavailable. Indeed, static faces that express high-intensity emotions provide enough emotional signals for the identification of the respective emotions, [53], [54], [55], [29]. The dynamic presentation of facial expressions would therefore seem to be more useful in ambiguous situations or for the identification of emotions by people with neurological or developmental disorders (brain damage or autism). For example, Harwood, Hall and Shinkfield [56] found that emotion recognition among subjects with mental retardation improved in response to moving facial expressions. Consistently with this idea, our results showed that the perception of movements in facial expressions increases, but does not change the nature of the emotional effects on time judgments, already observed with static images. Indeed, the static images of high emotional intensity (150 %) used in our study were sufficiently arousing to generate a significant lengthening effect.

The mechanisms involved in the processing of moving emotional facial expressions are complex and their effects on time must be further examined. Nevertheless, three main explanations can be proposed. Firstly, the increase in time distortions in response to facial movement could be due to the fact that this motion increases the intensity and arousal of perceived emotions, [57]. However, in our study, the rating of the arousal level induced by facial expressions did not vary between the dynamic and static displays. Secondly, as suggested by Matthews et al. [51] and Beckmann and Young [45], the motion could increase the amount of attention paid to stimuli, and therefore also to their emotional characteristics. Thirdly, it has been demonstrated that facial movements facilitate the mimicry of facial expressions and their internal simulation, [58], [59], [51], [28]. For instance, the differences between genuine and false smiles (spontaneous *vs.* deliberate) are detected better in dynamic than in static displays [58]. However, when the participants in a morphing task held a pen in their mouths that prevented them from mimicry, they could no longer detect the authenticity of smiles, [59]. Similarly, Effron, Niedenthal, Gil and Droit-Volet [60] showed that the lengthening effect observed in response to angry faces in a temporal bisection task disappeared when the participants could not perform facial mimicry because they were holding a pen in their mouths. It is thus possible that the moving faces might have facilitated the processes that accelerate the internal clock through the mimicry of perceived facial movements. Imaging studies conducted in the field of social neuroscience have shown that the judgment of emotions is associated with the activation of the motor and premotor cortex, [25]. As demonstrated by Adolphs and coworkers, the emotional and motor components of perceived facial expressions generate both a somatosensory and a motor representation of these facial expressions, [61], [62]. Subjective time distortions would consequently be an implicit indicator of individual adaptive abilities to attune to others' movements (actions) and/or of action readiness. This idea that the motor component of emotional expressions has a major influence on the perception of time is also consistent with the results of studies that have found time dilatations in response to photographs of body postures, [18], [63], [64]. For instance, Nather et al. [63] showed to their participants pictures of Edgar Degas' sculptures of a ballerina in different positions, and found that the presentation duration of a body position representing an expansive movement (movement of the great arabesque) was judged longer than that of a body posture with no movement (ballerina at rest). Within the framework of the theory of embodied time, [3], [18], [64], the authors explained their results in terms of the internal simulation of the body movements that underlie the perceived posture, along with its temporal properties, namely an internal clock that runs faster in the case of motor execution. This echoes Wittmann and Craig's theories on the activation of insula in temporal tasks and the role of proprioceptive information in the explicit experience of time, [65], [66], [67]. The direct experience or the reactivation of the experience (simulation) of other people's actions and movements would thus lie at the heart of time distortions in social contexts. However, further data are required if we are to validate this “embodied time” hypothesis and reject a simple attention-based hypothesis.

In conclusion, our results showed that time judgments are highly sensitive to movements in facial expressions as well as to the expressed emotions. Indeed, longer perceived durations were found for the dynamic faces and high-arousing emotional expressions than for the static faces and low-arousing facial expressions. Furthermore, our results showed that facial movements amplify the emotional effect of facial expressions on the perception of time. Dynamic emotional stimuli are thus interesting tools for examining time judgments and their variations in different social contexts. However, methodological precautions must be taken if we are to successfully identify the respective contributions of the dynamic structure of stimuli and their emotional characteristics.

## References

[1] Chambon M, Droit-Volet S, Niedenthal PM (2008) The effect of embodying the elderly on time perception. Journal of Experimental Social Psychology 44: 672–678.

[2] Conway LG (2004) Social contagion of time perception. Journal of Experimental Social Psychology 40: 113–120.

[3] Droit-Volet S, Gil S (2009) The time-emotion paradox. Journal of Philosophical Transactions of the Royal Society, B- Biological Sciences 364: 1943–1953. 10.1098/rstb.2009.0013 PMC2685815 19487196

[4] Damasio A (2000) The feeling of what happens: Body, Emotion, and consciousness. London: Random House.

[5] Frijda NH (1986) The emotions. Cambridge, MA: MIT press.

[6] Frijda NH (2007) The laws of emotion. Mahwath, NJ: Erlbaum.

[7] Kaiser S, Wehrle T, Schenkel K (2009) Expression faciale des emotions. In: Sander D, Scherer K, editors.Traité de psychologie des emotions. Paris: Dunod. pp. 109–157.

[8] Darwin C (1998) The expression of the emotions in man and animals. Oxford: Oxford University Press. [Original work published 1872].

[9] Lazarus RS (1991) Emotion and Adaptation. New York: Oxford University Press.

[10] Bar-Haim Y, Kerem A, Lamy D, Zakay D (2010) When time slows down: The influence of threat on time perception in anxiety. Cognition & Emotion 24: 255–263.

[11] Doi H, Shinohara K (2009) The perceived duration of emotional face is influenced by the gaze direction. Neuroscience Letter 457: 97–100. 10.1016/j.neulet.2009.04.004 19429171

[12] Droit-Volet S, Brunot S, Niedenthal P (2004) Perception of the duration of emotional events. Cognition and Emotion 18: 849–858.

[13] Droit-Volet S, Meck WH (2007) How emotions colour our time perception. Trends in Cognitive Sciences 1: 504–513. 10.1016/j.tics.2007.09.008 18023604

[14] Gil S, Droit-Volet S (2011) How do emotional facial expressions influence our perception of time? In: Masmoudi S, Yan Dai D, Naceur A, editors.Attention, Representation, and Human Performance: Integration of Cognition, Emotion and Motivation. London, UK: Psychology Press, Taylor & Francis.

[15] Tipples J (2008) Negative emotionality influences the effects of emotion on time perception. Emotion 8: : 127–131. 10.1037/1528-3542.8.1.127 18266523

[16] Tipples J (2011) When time stands still: Fear-specific modulation of temporal bias due to threat. Emotion 1: 74–80. 10.1037/a0022015 21401227

[17] Droit-Volet S (2013) Time Perception, Emotions and Mood Disorders. Journal of Physiology-Paris 107: 255–264. 10.1016/j.jphysparis.2013.03.005 23542546

[18] Droit-Volet S, Fayolle SL, Lamotte M, Gil S (2013) Time, emotion and the embodiment of timing. Timing and time perception 0: 1–30.

[19] Gil S, Droit-Volet S (2011) Time perception in response to ashamed faces in children and adults. Scandinavian Journal of Psychology 52: 138–145. 21265856 10.1111/j.1467-9450.2010.00858.x

[20] Ogden RS (2013) The effect of facial attractiveness on temporal perception. Cognition and Emotion 27: 1292–1304. 23410065 10.1080/02699931.2013.769426

[21] Gibbon J (1977) Scalar expectancy theory and Weber's law in animal timing. Psychological Review 84: : 279–325.

[22] Gibbon J, Church RM, Meck WH (1984) Scalar timing in memory. In: Gibbon J, Allan L., editors.Annals of the New York Academy of Sciences, 423: Timing and time perception.New York, US: New York Academy of Sciences. pp. 52–77. 10.1111/j.1749-6632.1984.tb23417.x 6588812

[23] Treisman M (1963) Temporal discrimination and the indifference interval: Implications for a model of the internal clock. Psychological Monographs 77: 1–13. 10.1037/h0093864 5877542

[24] Gil S, Niedenthal PM, Droit-Volet S (2007) Anger and time perception in children. Emotion 7: 219–225. 17352578 10.1037/1528-3542.7.1.219

[25] Decety J, Grèzes J (1999) Neural mechanism subserving the perceptions of human actions. Trends in Cognitive Sciences 3: 172–178. 10322473 10.1016/s1364-6613(99)01312-1

[26] Bassili JN (1979) Emotion recognition: The role of facial movement and the relative importance of upper and lower areas of the face. Journal of Personality & Social Psychology 37: 2049–2058. 521902 10.1037//0022-3514.37.11.2049

[27] Biele C, Grakowska A (2006) Sex differences in perception of emotion intensity in dynamic and static facial expressions. Experimental Brain Research 26: 1–6. 10.1007/s00221-005-0254-0 16628369

[28] Sato W, Yoshikawa S (2007) Spontaneous facial mimicry in response to dynamic facial expressions. Cognition 104: 1–18. 16780824 10.1016/j.cognition.2006.05.001

[29] Wehrle T, Kaiser S, Schmidt S, Scherer KR (2000) Studying the dynamics of emotional expression using synthesized facial muscle movements. Journal of Personality and Social Psychology 78: 105–119. 10653509 10.1037//0022-3514.78.1.105

[30] Krumhuber EG, Kappas A, Manstead ASR (2013) Affects of Dynamic Aspects of Facial Expressions: A Review. Emotion Review 5: 41–46.

[31] Lang PJ, Bradley MM, Cuthbert BN (2008) International Affective Picture System (IAPS): Affective Ratings of Pictures and Instruction Manual. Technical Report A- 8. Gainesville, FL: University of Florida.

[32] Ekman P, Friesen W (1976) Pictures of facial afffect. Palo Alto, CA, USA: Consulting Psychologist Press.

[33] Benson PJ, Perrett DI (1993) Extracting prototypical facial images from exemplars. Perception 22: 257–262. 8316513 10.1068/p220257

[34] Killeen P, Fetterman J, Bizo L (1997) Time's Causes. Time and Behaviour: Psychological and Neurobehavioural Analyses. In: Bradshaw CM, Szabadi E, editors.Time and behavior: Psychological and neurobehaviour analyses North-Holland: Elsevier Science Publishers. pp. 79–131.

[35] Allan LG (2002) The location and interpretation of the bisection point. The Quarterly Journal of Experimental Psychology 55: 43–60. 11900306 10.1080/02724990143000162

[36] Angrilli A, Cherubini P, Pavese A, Manfredini S (1997) The influence of affective factors on time perception. Perception & Psychophysics 59: 972–982. 9270369 10.3758/bf03205512

[37] Gil S, Droit-Volet S (2012) Emotional time distortions: The fundamental role of arousal. Cognition and Emotion 26: 847–862. 22296278 10.1080/02699931.2011.625401

[38] Grommet EK, Droit-Volet S, Gil S, Hemmes NS, Baker AH, et al (2010) Effects of a fear cue on time estimation in human observers. Behavioral Processes 86: 88–93. 10.1016/j.beproc.2010.10.003 20971168

[39] Lui MA, Penney TB, Schirmer A (2011) Emotion effects on timing: Attention versus pacemaker accounts. PlosOne, 6: , e21829. 10.1371/journal.pone.0021829 PMC3140483 21799749

[40] Shi Z, Jia L., & Müller, H J. (2012). Modulation of tactile duration judgments by emotional pictures. Frontiers in Integrative Neuroscience, doi:10.3389/fnint.2012.00024. 10.3389/fnint.2012.00024 PMC3358720 22654742

[41] Noulhiane M, Mella N, Samson S, Ragot R, Pouthas V (2007) How emotional auditory stimuli modulate time perception. Emotion 7: 697–704. 18039036 10.1037/1528-3542.7.4.697

[42] Mella N, Conty L, Pouthas V (2010) The role of physiological arousal in time perception: Psychophysiological evidence from an emotion regulation paradigm. Brain and Cognition 75: 182–187. 21145643 10.1016/j.bandc.2010.11.012

[43] Droit-Volet S, Ramos D, Bueno JLB, Bigand E (2013) Music, emotion and time estimation: The influence of subjective emotional valence and arousal? Frontiers, doi: 10.3389/fpsyg.2013.00417 10.3389/fpsyg.2013.00417 PMC3713348 23882233

[44] Vuilleumier P (2005) How brains beware: Neural mechanisms of emotional attention. Trends in Cognitive Sciences 9: 585–594. 16289871 10.1016/j.tics.2005.10.011

[45] Beckmann JS, Young ME (2009) Stimulus dynamics and temporal discrimination: Implications for pacemakers. Journal of Experimental Psychology: Animal Behavioral Processes 35: 525–537. 10.1037/a0015891 19839705

[46] Brown SW (1995) Time, change, and motion: The effects of stimulus movement on time perception. Perception & Psychophysics 57: 105–116. 7885802 10.3758/bf03211853

[47] Lhamon WT, Goldstone S (1975) Movement and the judged duration of visual targets. Bulletin of the Psychonomic Society 5: 53–54.

[48] Piaget P (1946) Le développement de la notion de temps chez l'enfant. Paris, France: PUF.

[49] Fraisse P (1967) Psychologie du temps. Paris, France: PUF.

[50] Matthews WJ (2011) How do changes in speed affect the perception of duration? Journal of Experimental Psychology: Human Perception & Performance 37: 1617–1627. 21517218 10.1037/a0022193

[51] Matthews WJ, Stewart N, Wearden JH (2011) Stimulus intensity and the perception of duration. Journal of Experimental Psychology: Human perception and performance 37: 303–313. 20731508 10.1037/a0019961

[52] Wang L, Yi Jiang Y (2012) Life motion signals lengthen perceived temporal duration. Proceedings of the National Academy of Sciences 109: 673–677. 10.1073/pnas.1115515109 PMC3306663 22215595

[53] Ambadar Z (2005) Schooler JW, Cohn JF (2005) Deciphering the enigmatic face: The importance of facial dynamics in interpreting subtle facial expressions. Psychological Science 16: 403–410. 15869701 10.1111/j.0956-7976.2005.01548.x

[54] Bould E, Morris N (2008) Role of motion signals in recognizing subtle facial expression of emotion. British Journal of Psychology 99: 167–189. 17535474 10.1348/000712607X206702

[55] Niedenthal PM, Brauer M, Halberstadt JB, Innes-Ker AH (2001) When did her smile drop? Facial mimicry and the influences of emotional state on the detection of change in emotional expression. Cognition and Emotion 15: 853–864.

[56] Harwood NK, Hall LJ, Shinkfield AJ (1999) Recognition of facial emotional expressions from moving and static displays by individual with mental retardation. American Journal of Mental Retardation 104: 270–278. 10349468 10.1352/0895-8017(1999)104<0270:ROFEEF>2.0.CO;2

[57] Krumhuber EG, Manstead ASR, Kappas A (2007) Temporal aspects of facial displays in person and expression perception: The effects of smile dynamics, head-tilt and gender. Journal of Nonverval Behavior 31: 39–56.

[58] Krumhuber EG, Manstead ASR (2009) Can Duchenne smiles be feigned? New evidence on felt and false smiles. Emotion 9: 807–820. 20001124 10.1037/a0017844

[59] Maringer M, Krumhuber EG, Fischer A, Niedenthal PM (2011) Beyond smile dynamics: Mimicry and beliefs in judgments of smiles. Emotion 11: 181–187. 21401238 10.1037/a0022596

[60] Effron D, Niedenthal P, Gil S, Droit-Volet S (2006) Embodied temporal perception of emotion. Emotion 6: 1–9. 16637745 10.1037/1528-3542.6.1.1

[61] Adolphs R (2002) Neural systems for recognizing emotion. Current Opinion in Neurobiology 12: 169–177. 12015233 10.1016/s0959-4388(02)00301-x

[62] Adolphs R, Damasio H, Tranel D, Cooper G, Damasio AR (2000) A role for somatosensory cortices in the visual recognition of emotion as revealed by 3-D lesion mapping. Journal of Neuroscience 20: 2683–2690. 10729349 10.1523/JNEUROSCI.20-07-02683.2000 PMC6772225

[63] Nather FC, Bueno JLO, Bigand E, Droit-Volet S (2011) Time changes with the embodiment of another's body posture. PlosOne 6: 1–7. 10.1371/journal.pone.0019818 PMC3103514 21637759

[64] Droit-Volet S (In press) What emotions tell us about Time. In: Llyod D, Arstila V., editors.Subjective Time: The philosophy, psychology, and neuroscience of temporality. Cambridge, MA: MIT Press.

[65] Craig AD (2009) Emotional moments across time: A possible neural basis for time perception in the anterior insula. Journal of Philosophical Transactions of the Royal Society, B- Biological Sciences 364: 1933–1942. 10.1098/rstb.2009.0008 PMC2685814 19487195

[66] Meissner K, Wittmann M (2011) Body signals, cardiac awareness, and the perception of time. Biological Psychology 86: 289–297. 21262314 10.1016/j.biopsycho.2011.01.001

[67] Wittmann M (2013) The inner sense of time: How the brain created a representation of duration. Nature Reviews Neuroscience 14: 217–223. 23403747 10.1038/nrn3452

